# MRI comparative study of diffuse midline glioma, H3 K27-altered and glioma in the midline without H3 K27-altered

**DOI:** 10.1186/s12883-022-03026-0

**Published:** 2022-12-22

**Authors:** Ji-ping Zhao, Xue-jun Liu, Hao-zhi Lin, Chun-xiao Cui, Ying-jie Yue, Song Gao, Hong-zhang Xu

**Affiliations:** 1 Department of Radiology, The Affiliated Hospital of Qingdao University, Qingdao, China; 2 Department of Stomatology, The Affiliated Hospital of Qingdao University, Qingdao, China; 3 Department of Radiology, Shidao People’s Hospital, Rongcheng, China

**Keywords:** Diffuse midline glioma, Magnetic resonance imaging, H3 K27-altered

## Abstract

**Purpose:**

The MRI features of Diffuse midline glioma, H3 K27-altered and glioma in the midline without H3 K27-altered were compared and analyzed, and the changes in the apparent diffusion coefficient (ADC) of the two groups were quantitatively analyzed.

**Methods:**

The MRI images of 35 patients with Diffuse midline gliomas, H3 K27-altered and gliomas in the midline without H3 K27-altered were analyzed retrospectively. The location, edge, signal, peritumoral edema and enhancement characteristics of the lesions were observed, and the changes in ADC values were analyzed.

**Results:**

In the H3 K27-altered group, 85.7% (12/14) of the tumors were located in the thalamus and brainstem compared with 28.6% (6/21) in the no H3 K27-altered group. In the H3 K27-altered group, for tumors only located in the midline area, only 14.3% (1/7) had irregular shapes and unclear boundaries, while for tumors also invaded the extramidline tissues 85.7% (6/7) had irregular shapes and unclear boundaries.The"basilar artery wrapped sign” was found in 6 patients with tumors located in the pons in the H3 K27-altered group, but none in the no H3 K27-altered group had this sign. In the H3 K27-altered group, only 14.3% (1/7) of the tumors confined to the midline area had small cystic degeneration and necrosis, while for tumors also invaded the extramidline tissues, 100% (7/7) of the tumors had cystic degeneration and necrosis, and the cystic degeneration and necrosis only located in the extramidline region of the tumor in 6 cases.A total of 78.6% (11/14) of tumors in the H3 K27-altered group showed mild to moderate enhancement, while 47.6% (10/21) of tumors in the no H3 K27-altered group showed mild to moderate enhancement. The average peritumoral edema index was 1.13 in the H3 K27-altered group and 1.75 in the no H3 K27-altered group. The average ADC value of tumor in the H3 K27-altered group was 7.83 × 10^− 4^ mm^2^/s, and the ratio to normal brain tissue was 0.844, while the values in the no H3 K27-altered group were 13.5 × 10^− 4^ mm^2^/s and 1.75, respectively.

**Conclusion:**

Compared with gliomas in the midline without H3 K27-altered, The MRI findings and ADC value of Diffuse midline gliomas, H3K27-altered have some characteristics, which can help improve the diagnosis and differential diagnosis.

In 2021, the World Health Organization (WHO) reclassified diffuse astrocytomas of the central nervous system (CNS). Diffuse midline glioma, H3 K27-altered had been redefined to reflect some newly discovered changes (e.g., EZHIP protein overexpression) in addition to the previously Diffuse midline glioma, H3 K27 mutations in the 2016 classification [1]. It is a high-grade glioma and often diffusely infiltrates the midline structure. There is a mutation at position 27 of the histone H3 gene, including histone H3.3 (H3F3A) and H3.1 (HIST1H3B) gene. This mutation leads to recurrent substitution of lysine to methionine, which reduces histone tail methylation, hinders glial differentiation, and then forms glioma [2–4]. Previous studies have shown that patients with gliomas with H3K27-altered have a poorer prognosis than those patients with gliomas without H3 K27-altered, with a 2-year survival rate of less than 10% [1, 5, 6]. Therefore, the accurate diagnosis before operation has important clinical significance. In this study, the MR images and ADC values of 14 patients with Diffuse midline gliomas, H3 K27-altered and 21 patients with gliomas in the midline without H3 K27-altered were analyzed retrospectively. The imaging features were analyzed, and the differences between them were summarized to improve the accuracy of the preoperative diagnosis of this disease.

## Materials and methods

### General information

Data from a total of 35 patients with midline gliomas from 2017 to 2019 were collected. The clinical data and MR images were studied retrospectively.Plain MRI scans and enhanced scans were performed in both groups. Diffusion weighted imaging (DWI) was performed in 24 patients with intracranial tumors, and the ADC values of the solid part of the tumor were measured by 3 experienced radiologists. Eleven tumors were located in the spinal cord, so DWI images were not performed. A total of 34 patients in the two groups underwent tumor resection, and 1 patient underwent intracranial tumor biopsy. Postoperative pathology and immunohistochemical detection of H3 K27-altered were performed.

### Inspection method

We used GE3.0T MRI and 32 channel head coil for scanning. Each scanning sequence and scanning parameters were as follows: T1WI (FSE sequence, TR: 1708ms, TE: 10ms, sagittal and axial position, slice thickness: 5.5 mm, spacing: 1.0 mm, NEX: 2), T2WI (fast FSE sequence, TR: 3580ms, TE: 104ms, horizontal axis, slice thickness: 5.5 mm, spacing: 1.0 m, NEX = 2), T2 flair (fast FSE, TR: 8402 ms, TE: 131 ms, horizontal axis, layer thickness: 5.5 mm, spacing: 1.0 mm, NEX: 2), DWI (EPI-SE, TR = 2120 ms, TE = 63 ms, the B values were 0 and 1000 s/mm, axis, slice thickness: 5.5 mm, spacing: 1.0 mm). Gd DTPA was selected as contrast agent for enhanced scanning. Axial, coronal and sagittal T1WI scans were performed, and the scanning parameters were the same as the above T1WI.

### Image analysis and pathological examination

We evaluated the position, shape, cystic change and necrosis of the tumor, as well as the edge of the lesion, the enhancement characteristics of the tumor, intratumoral hemorrhage and peritumoral edema on MRI.These characteristics were assessed by two observers. If the results are inconsistent, a senior doctor will join in, and the final result will be determined after discussion. The maximum diameter (A and B, respectively) on axial plane and the maximum height (C) on coronal plane of the tumor and its surrounding edema were measured. We used the formula (V = 4/3π × ABC) to calculate the edema and tumor volume (V edema + V tumor), and tumor volume (V tumor). The calculation formula of edema index (EI) is (V edema + V tumor)/V tumor. EI = 1 means no edema, 1 < EI < 1.5 means mild edema, 1.5 ≤ EI < 3 means moderate edema, and EI ≥ 3 means severe edema. The tumor specimens of all patients were examined by HE staining and immunohistochemistry. Immunohistochemistry included H3K27M mutation detection, using histone H3 mutant specific antibody (RM192, RevMAb Biosciences USA, Inc.). The sequencing reactions of Histone H3F3A and HIST1H3B/C were analyzed by direct sequencing of polymerase chain reaction–amplified products from tumor DNA.

### Statistical methods

Using SPSS 19.0 statistical software, the chi-square test was used to compare the differences in tumor location, shape and edge, internal cyst necrosis and intratumoral hemorrhage between the two groups, when *p* < 0.05, the difference is statistically significant. The peritumoral edema index and ADC value of the tumor were compared by independent sample t-tests, when *p* < 0.05, the difference is statistically significan.

## Results

Of the 35 patients with midline gliomas,14 patients with Diffuse midline gliomas, H3 K27-altered, aged 8–70 years, with a median of 19 years, and 21 patients with gliomas without H3 K27-altered, aged 7–62 years, with a median of 33 years.

### Location and morphology

**Fig. 1:**
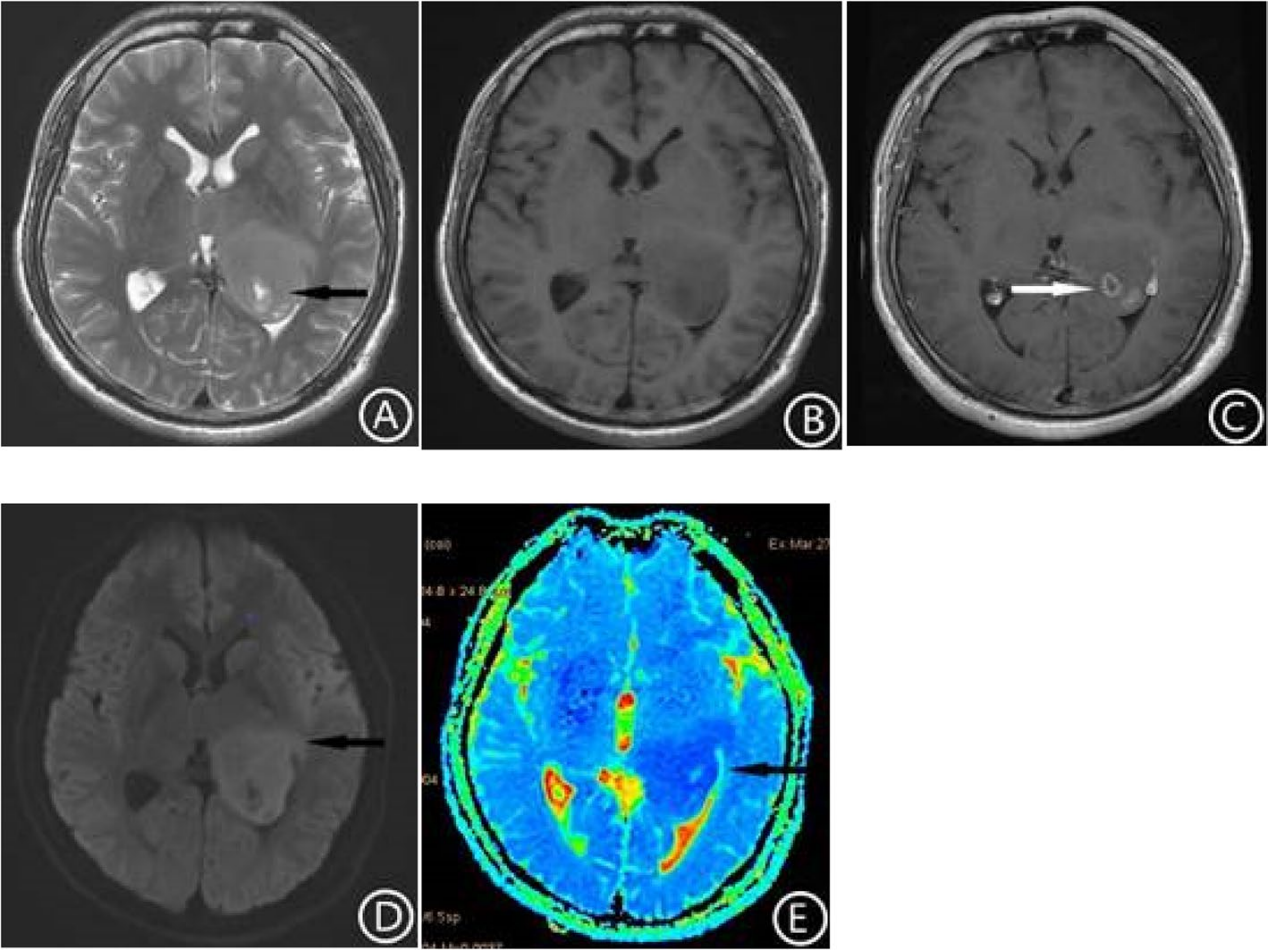
Brain MRI, DWI and ADC mapping in patients with Diffuse midline glioma, H3 K27-altered. The lesion was located in the left thalamus and invaded the left ventricle. The internal signal of the tumor was uneven, T2WI showed high signal intensity (**A**), and T1WI showed low signal intensity (**B**). Multiple cysts can be seen in the part of the tumor invading the lateral ventricle (**A**, arrow). There was no edema around the lesion. After enhancement, the tumor showed inhomogeneous enhancement, with circular enhancement (**C**, arrow). The tumor tissue showed high signal intensity on DWI. The boundary of the tumor was unclear and grew infiltratively along the lateral ventricular wall (**D**, arrow). On ADC mapping, the lesion showed low signal (**E**). The ADC value of the tumor tissue (6.97 × 10^− 4^ mm^2^/s) was significantly lower than that of the contralateral side (8.47 × 10^− 4^ mm^2^/s)

**Fig. 2:**
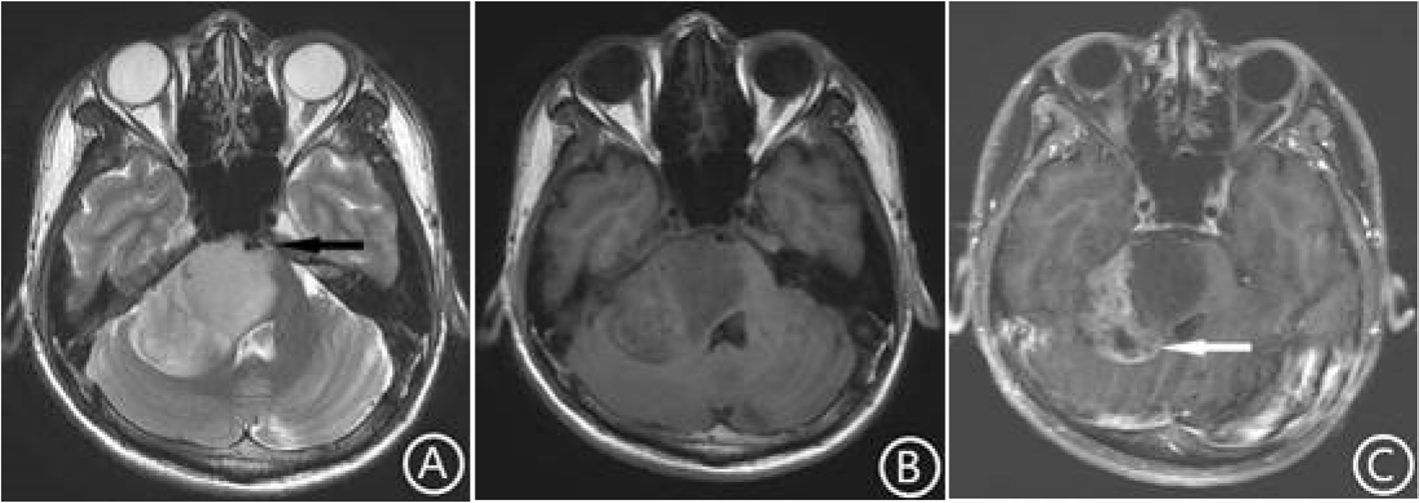
Brain MRI in patients with Diffuse midline glioma, H3 K27-altered. The lesion was located in the pons, right pontine arm and cerebellar hemisphere. The internal signal was uneven, T2WI showed a high signal (**A**), and T1WI showed a low signal (**B**). The boundary of the tumor was not clear, there was a “basilar artery wrapped sign” (**A**, arrow), and there was no edema around the tumor. After enhancement, the lesion showed inhomogeneous patchy enhancement and cystic necrosis (**C**, arrow) in the right cerebellar hemisphere and arm but no enhancement in the pons

**Fig. 3:**
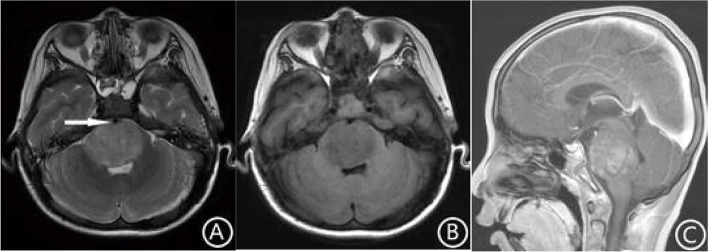
Brain MRI in patients with Diffuse midline glioma, H3K27-altered. The lesion was confined to the pons. The internal signal was uneven, T2WI showed a high signal (**A**), and T1WI showed a low signal (**B**). There was no cystic degeneration or necrosis in the lesion. The lesion had a clear boundary, there was a"basilar artery wrapped sign” (**A**, arrow), and there was no edema around the tumor. After enhancement, the tumor showed inhomogeneous patchy enhancement (**C**)

**Fig. 4:**
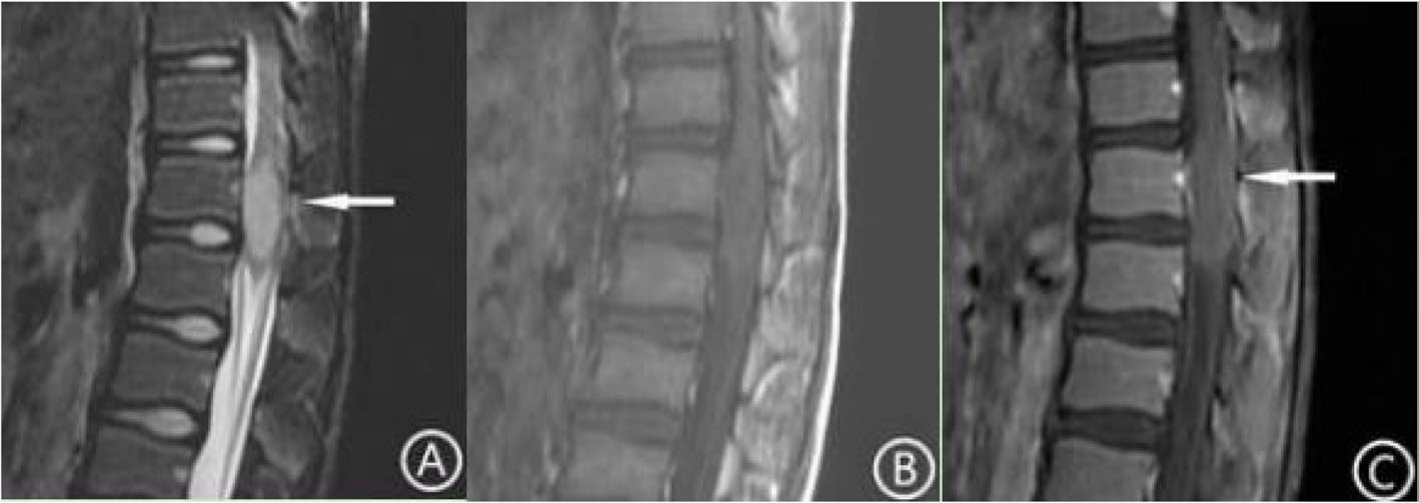
MR images of the thoracic vertebrae in patients with Diffuse midline glioma, H3K27-altered. The tumor was located in the conus medullaris. The internal signal was uniform, T2WI showed a high signal (**A**), and T1WI showed a low signal (**B**). There was no cystic necrosis inside the lesion (**A**, arrow) and no edema around the tumor. After enhancement, the tumor showed uniform mild enhancement (**C**, arrow)

**Fig. 5:**
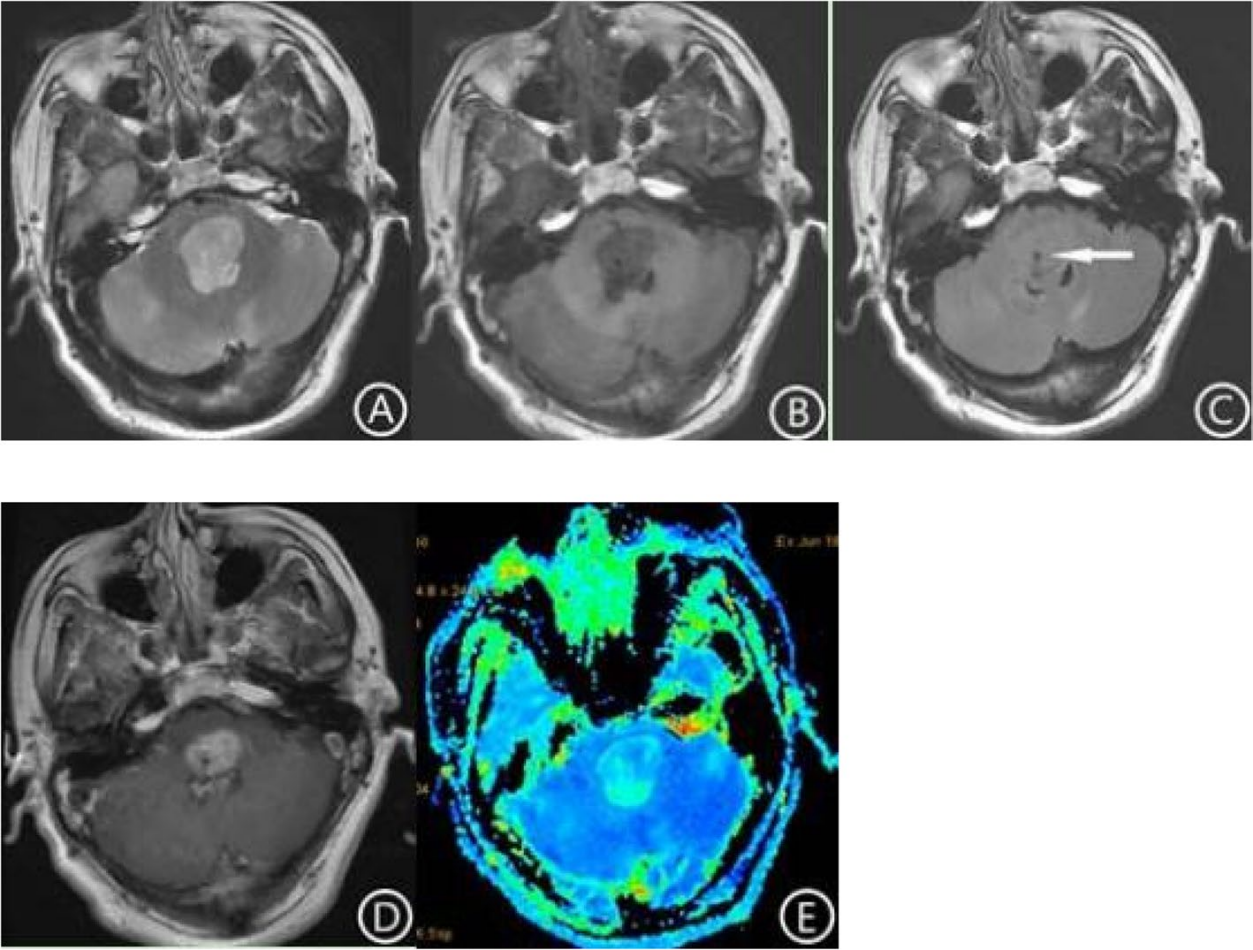
Brain MR images and ADC mapping in patients with glioma in the midline without H3 K27-altered. The tumor was located at the back of the pons and protruded into the fourth ventricle. The internal signal was uneven, T2WI showed a high signal (**A**), T1WI showed a low signal (**B**), and FLAIR showed an equal signal and cystic necrosis (**C**, arrow). There was no edema around the lesion. After enhancement, the tumor showed inhomogeneous and obvious enhancement (**D**). On ADC mapping, the lesion showed a high signal (**E**). The ADC value of the tumor tissue (9.65 × 10^− 4^ mm^2^/s) was higher than that of the surrounding normal brain tissue (8.95 × 10^− 4^ mm^2^/s)

The locations of the tumors in the two groups are shown in Table 1. In the H3 K27-altered group, 85.7% (12/14) of the tumors were located in the thalamus and brainstem (Figs. 1, 2 and 3), and 14.3% (2/14) were located in the spinal cord (Fig. 4). In the no H3 K27-altered group, 28.6% (6/21)of the tumors were located in the thalamus and brainstem (Fig. 5), 38.1%(8/21) in the spinal cord, and 33.3%(7/21) in cerebellar vermis. The chi-square test showed that the difference was statistically significant (χ^2^ = 11.667, *p* < 0.01). In the H3 K27-altered group, although the main body of the tumors were still located in the midline area, the tumors in 7 of the 14 patients also invaded the extramidline tissues, such as the pontine arm, cerebellar hemispheres and lateral ventricles (Figs. 1 and 2). While in the no H3 K27-altered group, the tumors in 11 of the 21 patients invaded the extramidline tissues.

**Table 1:** Clinical and MRI characteristics of Diffuse midline glioma, H3 K27-altered and glioma in the midline without H3 K27-altered

		All patients (*n* = 35)	Histone-H3K27 Mutant (*n* = 14)	Histone-H3K27 Wild-type (*n* = 21)	*P* Value
Median age (years)		29	19	33	< 0.05
Gender	Male	24	11	13	0.461
	Female	11	3	8	
The main part of tumor	Thalamus	5	4	1	< 0.01
	Pons	11	6	5	
	Vermis of cerebellum	7	0	7	
	Medulla oblongata	2	2	0	
	Cervical spinal cord	4	0	4	
	Thoracic spinal cord	1	0	1	
	Conus medullaris	5	2	3	
If involved the extramidline tissues	Limited to the midline	17	7	10	0.890
	Involved the extramidline tissues	18	7	11	
Mean survival time (years)		1.8	1.4	2.0	< 0.05
Cystic degeneration and necrosis	Yes	26	9	17	0.269
	None	9	5	4	
Intratumoral hemorrhage		8	4	4	0.511
Enhancement degree	Mild enhancement	8	5	3	< 0.05
	Moderate enhancement	13	6	7	
	Obvious enhancement	12	2	10	
	No enhancement	2	1	1	
Shape	Irregular	15	7	8	0.486
	Regular	20	7	13	
Edema	Mild edema	12	5	7	< 0.01
	Moderate edema	4	0	4	
	Severe edema	4	0	4	
	No edema	15	9	6	
Basilar artery wrapping sign	For tumors located in the pon (*n* = 11)	6/11	6/6	0/5	< 0.01

- data are the number of patients, age and time

In the H3 K27-altered group, the shape of the masses in 50% (7/14) of the patients was irregular, and the boundary was not clear, while in the no H3 K27-altered group, the shape of the mass was irregular, and the boundary was unclear in 38.1%(8/21) of the patients. The chi-square test showed that the difference was not statistically significant (χ^2^ = 0.486, *p* = 0.486). In the H3 K27-altered group, for tumors only located in the midline area, only 14.3% (1/7) had irregular shapes and unclear boundaries, while for tumors also invaded the extramidline tissues 85.7% (6/7) had irregular shapes and unclear boundaries (Figs. 1 and 2), the chi-square test showed that the difference was statistically significant (χ^2^ = 7.143, *p* < 0.01). In the no H3 K27-altered group, for tumors only located in the midline area, 50% (5/10) had irregular shapes and unclear boundaries, while for tumors also invaded the extramidline tissues 45.5% (5/11) had irregular shapes and unclear boundaries (Figs. 1 and 2),The chi-square test showed that there was no significant difference (χ^2^ = 0.043, *p* = 0.835). For the tumors located in the pons, all the 6 patients in the H3 K27-altered group showed the “basilar artery wrapped sign” (Figs. 2 and 3), while none of the 5 patients in the no H3 K27-altered group showed the “basilar artery wrapped sign”.

### Characteristics of MRI signals of the mass

Cystic degeneration and necrosis was found in 64.3% (9/14) of patients in the H3 K27-altered group and 81% (17/21) in the no H3 K27-altered group (Table 1). But the chi-square test showed that the difference was not statistically significant (χ^2^ = 1.222, *p* = 0.269). In the H3 K27-altered group, for tumors only located in the midline area, only 14.3% (1/7) had small cystic degeneration and necrosis, while for tumors also invaded the extramidline tissues, 100% (7/7) had cystic degeneration and necrosis (Figs. 1 and 2), and the cystic degeneration and necrosis only located in the extramidline region of the tumor in 6 cases. The chi-square test showed that the difference was statistically significant (χ^2^ = 10.500, *p* = 0.001). In the no H3 K27-altered group, for tumors only located in the midline area, 70% (7/10) had cystic degeneration and necrosis (Fig. 5), while for tumors also invaded the extramidline tissues, 90.9% (10/11) had cystic degeneration and necrosis. The chi-square test showed that the difference was not statistically significant (χ^2^ = 1.485, *p* = 0.223).

A total of 28.6% (4/14) of patients in the H3 K27-altered group had intratumoral hemorrhage, while 19.0% (4/21) of patients in the no H3 K27-altered group had intratumoral hemorrhage. In the H3 K27-altered group, 78.6% of the tumors had mild to moderate enhancement (Fig. 4C), and 14.3% had obvious enhancement. While in the no H3 K27-altered group, 47.6% (10/21) had mild to moderate enhancement (Fig. 5D), and 47.6% (10/21) had obvious enhancement. The chi-square test showed that the difference was significant (χ2 = 4.080, *p* < 0.05). There was mild edema or no edema around the tumor in the H3 K27-altered group (Table 1), and the average edema index was 1.13, while in the no H3 K27-altered group, the average edema index was 1.75. The independent sample t-test showed that the difference was statistically significant (t = 3.509, *p* < 0.01).

In the H3 K27-altered group, the tumors were showed high signal intensity on DWI, and low signal intensity on ADC map (Fig. 1). The average ADC value was 7.83 × 10^− 4^ mm^2^/s,. The average ratio of ADC value of tumor to normal brain tissue was 0.844. However, in the no H3 K27-altered group, the ADC map showed high signal intensity (Fig. 5E). The average ADC value was 13.5 × 10^− 4^ mm^2^/s, and the average ratio was 1.75. Independent sample t-tests showed that there were significant differences in the ADC values and its ratio between the two groups (t = 4.590, *p* < 0.01, t = 5.518, *p* < 0.01).

Postoperative follow-up showed that the average survival time of patients in the H3 K27-altered group was 1.4 years and that in the no H3 K27-altered group was 2.0years. Independent sample t-test showed that there was a significant difference between the two groups (t = 3.591, *p* < 0.05).

## Discussion

The prognosis of Diffuse midline gliomas, H3 K27-altered was poor, even if histopathology showed WHO grade II or grade III, which was the same as that of grade WHO IV gliomas. Therefore, Diffuse midline gliomas, H3 K27-altered are classified as WHO grade IV regardless of their histopathological manifestations [1].

### The age of the patients and location of the tumors

Diffuse midline glioma, H3K27-altered often occurs in children and adolescents, with few cases in middle-aged and elderly individuals, and there is no significant sex difference [7]. This group of patients was consistent with previous studies. Diffuse midline gliomas, H3 K27-altered are commonly found in the thalamus, brainstem and spinal cord [8]. Other rare sites include the third ventricle, hypothalamus, pineal region, cerebellar hemisphere and so on [9]. This group of patients showed that the location of the diffuse midline gliomas, H3 K27-altered was significantly different from gliomas in the midline without H3 K27-altered. This is consistent with previous studies. In this group of patients, brain tissue outside the midline structure was involved in both the H3 K27-altered group and the no H3 K27-altered group, indicating that diffuse midline glioma is not limited to only the midline area [10].

The clinical manifestation of diffuse midline glioma is related to its location but not to the type of tumor. Infratentorial tumors are often characterized by motor and sensory abnormalities, ataxia, cerebral neurological symptoms and so on. Supratentorial tumors often have symptoms such as increased intracranial pressure, hemiplegia, and blurred vision. The clinical manifestations of the Diffuse midline gliomas, H3 K27-altered and gliomas without H3 K27-altered included blurred vision, limb weakness, limb numbness, strabismus, headache, dizziness, facial paralysis, unstable walking and so on, [11].

### MR findings

In this group of patients, the Diffuse midline gliomas, H3K27-altered had regular shape and clear boundary when the tumors only located in the midline area, but the shape of the masses was irregular, and the boundary was not clear, when they invaded the extramidline tissues. However, gliomas without H3 K27-altered did not have this imaging feature. This feature has not been reported in previous studies, and its mechanism is not clear. It is speculated that this may be because Diffuse midline gliomas, H3K27-altered originate from the midline, so most of the gliomas confined to the midline are in the early stage or are weakly invasive, while the gliomas involving structures outside the midline are mostly in the late stage or have strong infiltration. This needs to be further studied. In this group, the “basilar artery wrapped sign” was only found in patients with Diffuse midline gliomas, H3 K27-altered. This sign is most common in high-grade gliomas located in the pons [12]. Therefore, if there is a “basilar artery wrapped sign”, it indicates that the tumor has a high degree of malignancy, and the possibility of Diffuse midline gliomas, H3 K27-altered should be considered.

Similar to that of other high-grade gliomas, the signal intensity of most Diffuse midline gliomas, H3 K27-altered is often uneven. The cystic part of the tumor showed obvious low signal intensity on T1WI and high signal intensity on T2WI, while the solid part of the tumor showed slightly low signal intensity on T1WI and slightly high signal intensity on T2WI. The tumor is prone to cyst degeneration, necrosis and hemorrhage [9], which may be related to the rapid growth of the tumor or to ischemia, hypoxia or other reasons. we found that Diffuse midline gliomas, H3 K27-altered were prone to cyst degeneration and necrosis as gliomas without H3 K27-altered. We found that the Diffuse midline gliomas, H3K27-altered which only located in the midline area had less cyst degeneration and necrosis, but when they invaded the extramidline tissues, the tumors had more cystic degeneration and necrosis, and the cystic degeneration and necrosis always only located in the extramidline region of the tumor. The reasons for this phenomenon need to be further studied.

After enhancement, the enhancement range of diffuse midline gliomas is different, which can only be shown as spot enhancement, circular enhancement, and obvious enhancement in large patches [9]. The enhancement degree of Diffuse midline gliomas, H3K27-altered was lower than that of no H3 K27-altered midline glioma. This is different from the enhancement of high-grade gliomas, which are often significantly enhanced [13]. The peritumoral edema of Diffuse midline gliomas, H3K27-altered was not obvious, and some did not have edema at all. This may be because the tumor is classified as WHO grade IV, but the histopathological features of some tumors are the same as those of WHO grade I and II tumors, and peritumoral edema is closely related to the pathological classification of tumors [14]. The vascular endothelium and intercellular connections of low-grade gliomas are relatively close, while the endothelial cells of high-grade gliomas are dysplastic and loosely connected; the higher the malignant degree of tumor cells is, the more immune and inflammatory factors secreted, thus aggravating brain edema. At the same time, high-grade gliomas grow faster, peritumoral edema will further oppress the surrounding brain tissue, and the venous reflux of the peritumoral tissue will be blocked, thus aggravating the degree of peritumoral edema [15, 16]. This group of patients showed that the peritumoral edema in the Diffuse midline gliomas, H3 K27-altered was mild or there was not edema, and the degree of peritumoral edema was significantly milder than that of gliomas without H3K27-altered, this was consistent with previous studies.

DWI can evaluate tumor tissue structure from the microscopic level, and the ADC value can better reflect the degree of tumor cell density and cell edema, which is highly consistent with the malignant degree of tumors, so it can predict the glioma grade [17]. we found that the ADC value of the tumor and its ratio to the normal brain tissue of Diffuse midline gliomas, H3 K27-altered were lower than those of gliomas without H3K27-altered. This reflects the dense cells, high nuclear/cytoplasmic ratio and high degree of malignancy of Diffuse midline gliomas, H3 K27-altered, which is in accordance with the histological characteristics of high-grade gliomas. Therefore, the ADC value can be used as a reference index for the diagnosis of Diffuse midline gliomas, H3 K27-altered.

In conclusion, for patients with gliomas in the midline area, those with H3K27-altered are younger in age. Compared with gliomas without H3 K27-altered, Diffuse midline gliomas, H3K27-altered are more likely to occur in the thalamus and brainstem. When the Diffuse midline gliomas, H3K27-altered only located in the midline area,,they often have regular shapes,, clear boundaries, and less cyst degeneration and necrosis. But when they invaded the extramidline tissues, the tumors often have irregular shape and unclear boundary and have more cystic degeneration and necrosis, in addition, the cystic degeneration and necrosis always only located in the extramidline region of the tumor. However, gliomas without H3 K27-altered did not have such imaging feature. Compared with gliomas in the midline without H3 K27-altered, H3K27M-mutant diffuse midline gliomas have less peripheral edema and less enhancement. The “basilar artery wrapped sign” is often seen in H3K27M-mutant midline gliomas located in the pons. The ADC value is helpful for preoperative tumor grading,

### Treatment and prognosis

At present, the treatment of Diffuse midline gliomas, H3K27-altered is mainly surgery, radiotherapy and chemotherapy. In recent years, there have been an increasing number of studies on gene targeting therapy, and some of these approaches have been used in the clinic [18], Some studies have shown that ONC201, a small molecule selective antagonist of dopamine receptor D2/3 (DRD2/3), may be effective for Diffuse midline gliomas, H3K27-altered [19]. The prognosis of patients with Diffuse midline gliomas, H3K27-altered is poorer than that of patients with glioma without H3 K27-altered [20]. This group of patients also showed that the survival time of Diffuse midline gliomas, H3K27-altered was significantly shorter than that of gliomas without H3 K27-altered, which was consistent with previous studies. Feng et al. [21] found that the prognosis of Diffuse midline gliomas, H3 K27-altered in different anatomic sites was different, and the prognosis of patients with Diffuse midline gliomas, H3K27-altered in the brainstem was worse than that in those with gliomas in the thalamus. This feature was not found in this group of patients, which may be related to the small sample size. However, Karremann et al. [22] found that H3K27M gene mutation was the only factor for poor prognosis in Diffuse midline gliomas, H3K27-altered and had nothing to do with the location, tumor grade or extent of involvement. Therefore, some scholars suggest that H3K27M mutation detection should be performed for all gliomas in the midline region in addition to histopathology and routine immunohistochemical detection [23].

Limitations of this study:The number of cases in this study is relatively small, and some imaging features still need to be verified by large samples of cases.Some features,like the degree of enhancement, were assessed by the observer subjectively. Therefore, there may be some slight inaccuracies. The volume of tumors was calculated by formula, the automatic method is not used. Although this method is convenient for clinical application, its accuracy is not as good as the automatic method.

## Conclusion

Compared with gliomas in the midline without H3 K27-altered, The MRI findings and ADC value of Diffuse midline gliomas, H3K27-altered have some characteristics, which can help improve the diagnosis and differential diagnosis. However, based on the imaging findings alone, it is difficult to make an accurate preoperative diagnosis for Diffuse midline gliomas, H3K27-altered. Immunohistochemical H3K27M detection and gene detection are the final criteria to determine the occurrence of H3K27M gene mutations.

## Data Availability

All data generated or analysed during this study are included in this published article.

## Abbreviations

ADC: apparent diffusion coefficient
CNS: central nervous system
WHO: World Health Organization
DWI: diffusion weighted imaging
